# Rationalizing Sequence and Conformational Effects on the Guanine Oxidation in Different DNA Conformations

**DOI:** 10.1021/acs.jpcb.2c02391

**Published:** 2022-06-07

**Authors:** Alessandro Nicola Nardi, Alessio Olivieri, Marco D’Abramo

**Affiliations:** Department of Chemistry, Sapienza University of Rome, Rome, Italy 00185

## Abstract

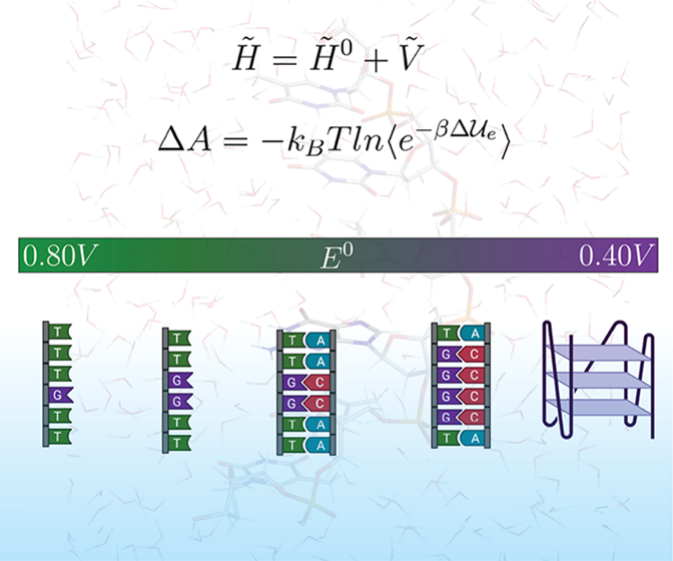

The effect of the environment on the guanine redox potential is studied by means of a theoretical–computational approach. Our data, in agreement with previous experimental findings, clearly show that the presence of consecutive guanine bases in both single- and double-stranded DNA oligomers lowers their reduction potential. Such an effect is even more marked when a G-rich quadruplex is considered, where the oxidized form of guanine is particularly stabilized. To the best of our knowledge, this is the first computational study reporting on a quantitative estimate of the dependence of the guanine redox potential on sequence and conformational effects in complex DNA molecules, ranging from single-stranded DNA to G-quadruplex.

## Introduction

Deoxyribonucleic acid (DNA) is the molecule used by living organisms to store the precious information needed to survive. DNA is constantly exposed to oxidant agents, both endogenous and exogenous, such as reactive oxygen species^1^ (ROS) and ionizing radiations.^2^ The events responsible for the DNA oxidation could lead to mutagenesis, carcinogenesis, and aging-related processes.^3,4^ DNA oxidative damage can cause loss and/or corruption of information retained in living organisms.

In addition, charge transfer along the double strand, which makes it a molecular wire,^5,6^ is supposed to play a decisive role in DNA repair mechanisms.^7^

To investigate the DNA oxidation process, a deep knowledge of its electronic properties in realistic environments and conformations is needed. As such, efforts in the estimation of the oxidation potentials of native and mutated nucleic acids have been made. However, from an experimental viewpoint, the measurement of the oxidation potentials of the building blocks of nucleic acids in solution, e.g., (2′-deoxy)nucleosides and (2′-deoxy)nucleotides, is partially hindered by low solubility, partial irreversible nature of the electrode oxidation reaction, and pH dependent electrochemical potential.^8−10^ For these reasons, theoretical–computational approaches are particularly useful because they are not limited by those experimental difficulties.^11−23^ Nevertheless, in these types of approaches, other complications exist: that is, the huge number of degrees of freedom in a system of nucleic acids in solution prohibits the quantum mechanical treatment of the entire system. To overcome this limitation, the effect of the environment on the nucleobase electronic properties is often treated by means of dielectric continuum models^24−26^ or by splitting the system into the QM part, where the electronic properties are explicitly calculated, and the MM part, which accounts for the effect of the environment on the QM region.^27,28^

In line with the latter, in the present study we made use of a QM/MM approach, the Perturbed Matrix Method^29−31^ (PMM), which blends the extended sampling provided by classical molecular dynamics simulations and quantum mechanical calculations, in order to evaluate the thermodynamics properties, i.e., the redox potentials, of guanine in complex DNA molecules.

The guanine was selected because in several experimental and computation works,^8,10,16,32^ wheresuch a nucleobase was found to be the easiest to oxidize. It was also found that sequences containing two or more adjacent guanine bases show a higher propensity to oxidation than a guanine base alone in solution or when no consecutive guanines are present along the DNA strand.^33−35^ A possible explanation concerns the delocalization of the positive charge over two (or more) bases.^36,37^ However, it was also observed that solvation favors the (electronic) hole confinement to one or few nucleobases.^20,38−40^ Computational and experimental (ESR spectroscopy) works agree that an electronic hole in 5′-GG-3′ and 5′-GGG-3′ sequences tends to localize to a single guanine site.^41−43^ To better understand the oxidation properties of guanine in realistic contexts, we applied the PMM–MD method to estimate the reduction potential of the guanine base cation in single- and double-stranded oligonucleotides and in a G-quadruplex structure in solution to investigate sequence, conformational, and environmental effects on the thermodynamics of the oxidation of such a nucleobase.

## Theory

### Perturbed Matrix Method

The MD–PMM is a quantum/classical hybrid method based on the combination of high-level quantum theory electronic calculations and molecular dynamics simulations. Its ability to treat complex systems in order to obtain thermodynamic and kinetic properties has been demonstrated.^12,44−47^

The method is based on the partition of the system into a region, in which the process of interest occurs, treated quantum mechanically (termed as the quantum center, QC; in our case, the redox center), and the effect of the environment is included as a semiclassical electrostatic perturbation, generated by the environmental atomic charges, for each configuration explored in the MD simulation.

Once the electronic properties of the QC in the gas phase are calculated, it is possible to construct the perturbed Hamiltonian matrix, :
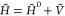
1expressed as the sum of the unperturbed Hamiltonian matrix, , and the perturbation matrix, . The elements of the perturbation matrix are obtained by
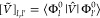
2where  and  are the unperturbed (gas-phase) eigenfunctions of the QC.  is the perturbation operator, which can be expressed by the Taylor series expansion (truncated at the first-order term) of the electrostatic potential around each *n*th atom belonging to the QC:
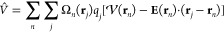
3The sum over *j* runs over all the QC nuclei and electrons, each one with its charge *q*_*j*_. In the first-order term, . Ω_*n*_ is a step function being null outside and unity inside the *n*th atomic region. This atom-based expansion is performed only for the diagonal elements; for the off-diagonal elements a perturbation operator expansion within the dipolar approximation is used:
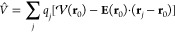
4with **r**_0_ being the coordinates of the QC center of mass.

The perturbed Hamiltonian matrix for the QC is constructed and diagonalized for each configuration sampled by MD, giving a trajectory of eigenvectors describing the perturbed eigenstates of the QC, nestled in the complex system, and the relative eigenvalues, i.e., the perturbed energies.

In the present case, we are interested in the evaluation of the ground-state energies of the redox center, i.e., the nucleobase, in the reduced (neutral, B) and in the oxidized (radical-cation, B^·+^) form in order to obtain the free energy of the one-electron reduction reaction (B^·+^ + e^–^ → B) and the reduction potentials of the oxidized nucleobases in single- and double-stranded oligonucleotides, a double-stranded DNA, and a G-quadruplex structure.

### Helmholtz Free Energy

The Helmholtz free energy change, Δ*A*, associated with the reduction reaction can be expressed as
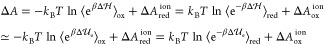
5where  is the QC environment whole energy change upon oxidation, with  being the corresponding QC perturbed electronic ground-state energy change. The electronic energy change is obtained at each classical configuration, relaxing the quantum nuclear degrees of freedom, and thus approximates the vibronic ground-state energy change (and hence  is the adiabatic ionization energy). The angle bracket subscripts ox and red indicate that both the energy change and the averaging are obtained in either the oxidized or reduced ensemble, respectively, each involving its own ionic condition. In the hypothesis of negligible environment internal energy change associated with the QC reaction (in the case of MD force fields and assuming the environment electronic state is independent of the QC oxidation state and is exactly zero), the approximation  holds.

 is the relaxation free energy for the reduced species due to the ox → red ionic environment transition, and  is the relaxation free energy for the oxidized species due to the red → ox ionic environment transition. Considering , the following can be written:
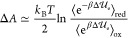
6This equation is the one used for the evaluation of the free energy change upon redox reaction. The perturbed electronic ground-state energy change and the ensemble averages are evaluated via the MD–PMM approach.

## Methods

In this case the QC coincides with the nucleobase in the substrate of interest. Hence, as indicated in the previous section, the gas-phase properties of the nucleobases are needed for the application of the PMM method.

For this purpose we optimized the geometry of the nucleobases, taken from the work of Psciuk et al.^16^ using the density functional theory (DFT) with the B3LYP functional and the 6-311++G(2d,2p) basis set, for the neutral and radical cation state. The unperturbed electronic properties of the ground state and the first seven excited states were calculated at the DFT and TD-DFT level of theory using the B3LYP functional and the 6-311++G(2d,2p) basis set. All the calculation were made using Gaussian 16^48^ and Dalton^49^ software. The calculated (B3LYP/6-311++G(2d,2p)) adiabatic ionization energy for the guanine base in the gas phase is 7.65 eV.

MD simulations were carried out for the single-stranded oligonucleotides ss-5′-d(TTTGTT)-3′ (ss-HG1), ss-5′-d(TTGGTT)-3′ (ss-HG2), and ss-5′-d(TGGGGT)-3′ (ss-HG4) and the corresponding double-stranded DNA ds-5′-d(TTTGTT)-3′ (ds-HG1), ds-5′-d(TTGGTT)-3′ (ds-HG2), and ds-5′-d(TGGGGT)-3′ (ds-HG4) in water both in the neutral and radical cation state at the site of the base involved in the redox process. Each oligonucleotide was placed in a cubic box with an edge of 4.873 nm filled with 3800 SPC^50^ (simple point charge) water molecules and a number of Na^+^ ions to achieve the system electroneutrality.

Additional MD simulations were carried out for a 12-base pair DNA fragment: ds-5′-d(CGTATGGGTACG)-3′ (ds-DG3) in water both in the neutral and radical cation state at the site of the base involved in the redox process. The molecule was placed in a cubic box with an edge of 6.745 nm and solvated by 10 013 SPC water molecules and 22 (21) Na^+^ ions in the reduced (oxidized) ensemble.

The substrates were selected from the work of Capobianco et al.^35^ to provide a tight comparison with experimental data. In that work, the guanine redox potentials were measured by means of differential pulse voltammetry (DPV) and reported against the  redox couple, considered as a quasi-reference electrode.^35^ Due to possible inaccuracies due to (i) the use of quasi-reference electrodes and (ii) dealing with irreversible processes,^51^ a quantitative comparison with the experimental data is discussed in terms of the shift (indicated by the symbol Δ) of the reduction potentials with respect to the ss-HG1, which is used as a reference.

Lastly, a simulation of a parallel G-quadruplex structure was carried out (7KLP in PDB^52^) with the sequence 5′-d(AGGG(TTAGGG)_3_)-3′ in a cubic box of 6.543 nm, filled with 9355 SPC water molecules and 21 (20) K^+^ ions in the neutral (oxidized) ensemble. Several MD simulations in the oxidized ensemble of the same system were performed charging in turn different guanines positive charge residing at different sites (guanine sites).

The temperature was kept constant, via the velocity rescaling with stochastic term algorithm,^53^ at 278 K for oligonucleotides and at 300 K for the dodecamer ds-DNA and the G-quadruplex. The simulations time length was 100 ns, and a time step of 2 fs was used. The volume was kept constant.

All the molecular dynamics simulations were made using the Gromacs software package^54^ and AMBER99 force field.^55^ For the simulations of the DNA containing nucleobases radical cations (i.e. simulations in the oxidized ensembles), the atomic partial charges were estimated by the same procedure used for the estimation of the parameters in the AMBER force field.^55^

For the calculation of one-electron reduction potentials of the nucleobases radical cations, the Helmholtz free energy associated with the B^·+^ + e^–^ → B process was used (calculated as reported in the Theory section):
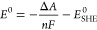
7where *F* is the Faraday constant and *n* is the number of electrons involved in the reaction. The value of the standard hydrogen electrode potential  was taken from the literature (4.281 V).^56^ The statistical errors were estimated by calculating the mean values of the observable in different subparts of the trajectory and evaluating the standard error. The convergence of the Helmholtz free energy, and thus of the reduction potential, was checked for each system by calculating its value at an increasing number of frames (see Figure S3). Note that our results are discussed in terms of guanine oxidation but reporting cationic guanine standard reduction potentials *E*^0^ to provide values directly comparable to literature.

## Results and Discussion

**Figure 1:**
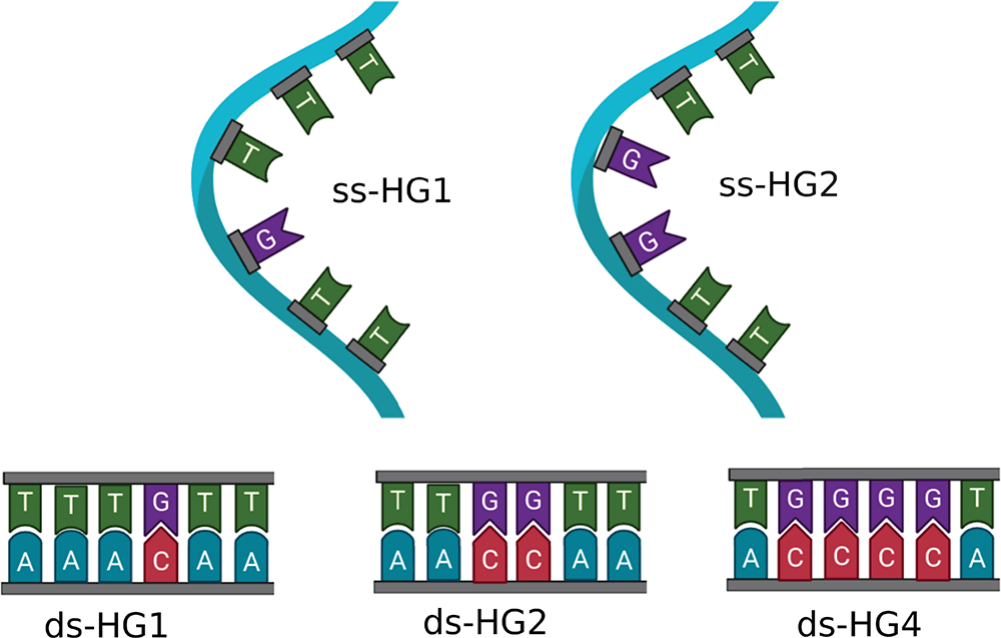
Schematic representation of the simulated single- and double-stranded DNA.

We calculated the cationic guanine redox potentials in two oligonucleotides: ss-5′-d(TTTGTT)-3′ (ss-HG1) and ds-5′-d(TTTGTT)-3′ (ds-HG1) (see Figure 1). These constructs were chosen because of the availability of experimental data obtained by differential pulse voltammetry measurements.^35^

**Figure 2:**
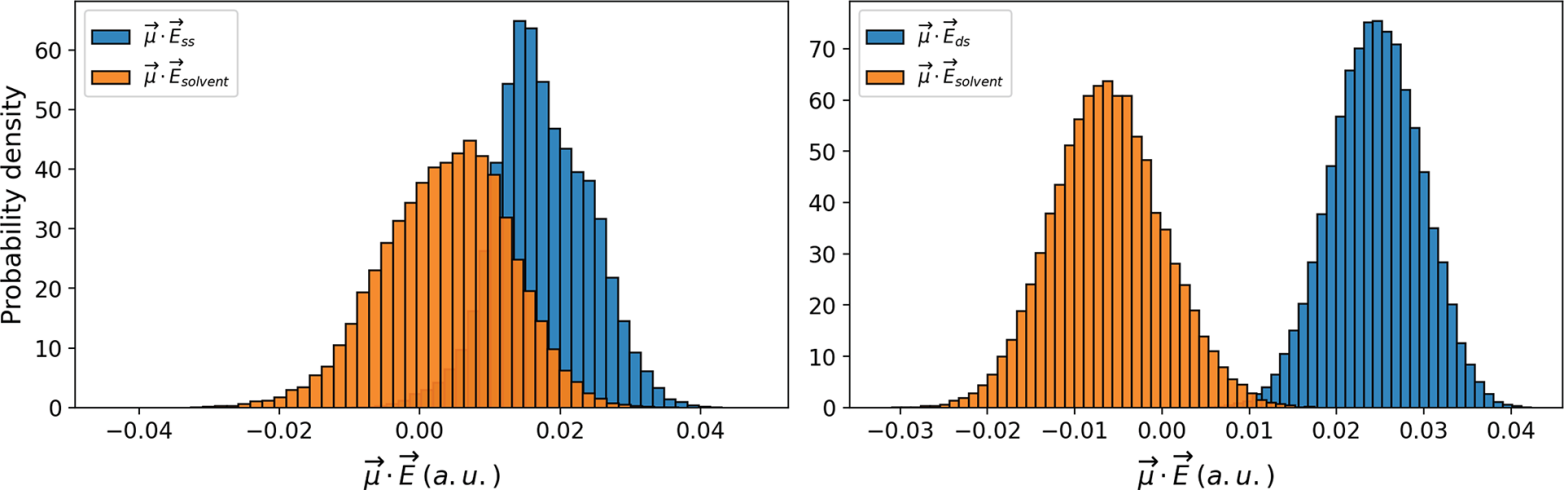
Analysis of the perturbation felt by the guanine in ss-HG1 and ds-HG1.

Using the guanine base as the QC in both cases, the estimated reduction potentials (*E*^0^) are 0.80 and 0.67 V (vs SHE) in single- and double-strands, respectively. Considering the value of the reduction potentials in the gas phase of a single guanine base (1.25 V), in both systems the perturbing environment favors the guanine oxidation, although to a different extent. The difference in the *E*^0^ values between single-stranded DNA (ss-DNA) and double-stranded DNA (ds-DNA) can be explained by the analysis of the guanine (perturbed) energies as obtained by the PMM–MD approach. In fact, the ionized state of the guanine is more stabilized by the environment in ds-DNA than in ss-DNA. An analysis of the perturbation as provided by the electric field felt by the QC shows that, in both systems, the rest of the DNA bases lead to a stabilizing contribution to the energy of the guanine in the ionized state (as calculated by , where  is the electric dipole moment of the ionized guanine base and  is the electric field generated by the inhomogeneous atomic–molecular environment), being higher in the double-stranded system (see Figure 2). The destabilizing effect of the solvent, which is more structured in ds-HG1, does not compensate the perturbation due to the DNA, thus resulting in the lower value of *E*^0^ in ds-HG1 with respect to ss-HG1.

The convergence of Δ*A* (and thus of *E*^0^) in both the neutral and oxidized ensemble was checked by a sensitivity analysis (see Figure S3).

The effect of the presence of two consecutive guanine bases on their redox potentials was investigated considering the two oligonucleotides ss-5′-d(TTGGTT)-3′ (ss-HG2) and ds-5′-d(TTGGTT)-3′ (ds-HG2). In these systems, a guanine replaces the third thymine of the two systems discussed above (ss-HG1 and ds-HG1).

In these cases, the guanine at the 5′ terminus was selected as the QC, whereas the guanine at the 3′ end acts as a part of the perturbing environment. The choice to consider the QC as formed by a single guanine base was motivated by the analysis of the charge distribution of an ionized guanine dimer. A dimer of guanine in cationic form was built using the optimized geometry of two isolated guanine bases placed in the same relative position as if they were in an ideal B-DNA strand (see Figure S2). The charge distribution of the dimer in the gas phase and in water as calculated at different levels of theory shows that solvation—as modeled by the dielectric continuum model—induces a charge localization on a single guanine base (see Table S1), in agreement with previous works.^20,38,39^ As such, it is reasonable to consider the QC as being formed by a single guanine base, i.e., assuming the localization of the positive charge on a single guanine.

The reduction potentials of the oxidized guanine bases in ss-HG2 and ds-HG2 are indistinguishable, being 0.69 V (vs SHE) for both the substrates.

Contrary to the hexamers ss-HG1 and ds-HG1, in ss-HG2 and ds-HG2 the presence of the complementary strand does not modify the reduction potential. This result reproduces the experimental findings^35^ at a quantitative level (see Table 1).

**Table 1:** Calculated Values of the Cationic Guanine Reduction Potentials in ss-DNA and ds-DNA^a^

system	*T* (K)	*E*^0^(*V*),^b^^,^^d^ PMM	Δ(*V*), PMM	Δ(*V*),^c^ exp.^35^
ss-HG1	278	0.80	0.00	0.00
ds-HG1	278	0.67	0.13	
ss-HG2	278	0.69	0.11	0.10
ss-HG2	300	0.83	–0.03	0.00
ds-HG2	278	0.69	0.11	0.11
ds-HG4	278	0.55	0.25	0.20

a Δ indicates the difference in the reduction potential with respect to ss-HG1; both calculated and experimental values are reported.

b Values are reported against SHE.

c Due to the use of a quasi-reference electrode, only the guanine reduction potential shifts with respect to ss-HG1 are reported.

d The estimated standard error on the calculated reduction potentials is ±0.04 V.

When ds-DNA with two additional guanine bases is considered, as in ds-5′-d(TGGGGT)-3′ (ds-HG4), a further decrease of the *E*^0^ of the guanine was observed with respect to the other hexamers considered before (see Table 1). The comparison between our results and the available experimental data, clearly showing the remarkable accuracy of our approach in all the systems considered, indicates that the effect of adding adjacent guanine bases from 1 to 4 leads to a decrease in the reduction potential of the ionized guanine base in the strand.

To shed light on the effect of the DNA conformation, and in particular of the nucleobase stacking, the reduction potential of the cationic form of guanine in the ss-HG2 system was calculated at different temperatures, i.e., 278 and 300 K. This was motivated by the results of an experimental work^35^ where the same value of the guanine oxidation potential was observed—at 300 K—in two single-stranded DNA, one containing a single guanine base (ss-HG1) and one containing a couple of consecutive guanine bases (ss-HG2). Such an effect was explained by the absence of stacking interactions along the strand. In fact, the measurement of the oxidation potential at a lower temperature (278 K), favoring the stacking interactions, showed the expected decrease of the reduction potential, i.e., 0.76 vs 0.66 V.^35^ Indeed, by performing an additional MD simulation of the ss-HG2 system at 300 K, we observe a very similar decrease of the *E*^0^ (0.69 V) with respect to the system at 300 K (0.83 V). The analysis of the stacking interactions in ss-HG2 as provided by the MD simulations at 278 and 300 K shows, as expected, that the fluctuations of the typical DNA base pair parameters are remarkably higher at 300 K with respect to 278 K (see Table S2 in the Supporting Information). These results substantiate the hypothesis, previously used to rationalize the experimental data where an atomic-based interpretation was attempted,^35^ that the presence of two consecutive guanines favors their oxidation as long as a proper, ”well-stacked” interaction between these nucleobases occurs.

To gain further insight into the effect of consecutive guanines on the redox potential, we also calculated the redox potential of the guanine base in ds-HG1 and ds-HG2 without the perturbing contribution of the neighbor nucleobase in the 5′ direction. That is, we removed the effect of the neighbor thymine in ds-HG1 and guanine in ds-HG2. These results, summarized in Table S3 in the Supporting Information, show that the reduction potential increases more in ds-HG2 than in ds-HG1 with respect to their corresponding redox potentials obtained considering the complete perturbation of the environment (see Table 1). Therefore, the decrease of the reduction potential due to the presence of the guanine stack is due to its direct effect leading to the stabilization of the guanine in its radical cationic form.

The effect of the sequence on the guanine oxidation potential was also investigated in a longer, double-stranded DNA dodecamer ds-5′-d(CGTATGGGTACG)-3′ (ds-DG3). In agreement with experimental results^35^ (*E*^0^ = 0.61 *V*), we estimated *E*^0^ = 0.75 V and *E*^0^ = 0.67 V (vs SHE) for the first and the central guanine bases, respectively. These data, confirming the stabilizing effect on the guanine oxidation potential due to the presence of consecutive guanines along the DNA strand, suggest that the limited number of nucleobases present in the hexamers does not affect the previous interpretation of the results.

**Figure 3:**
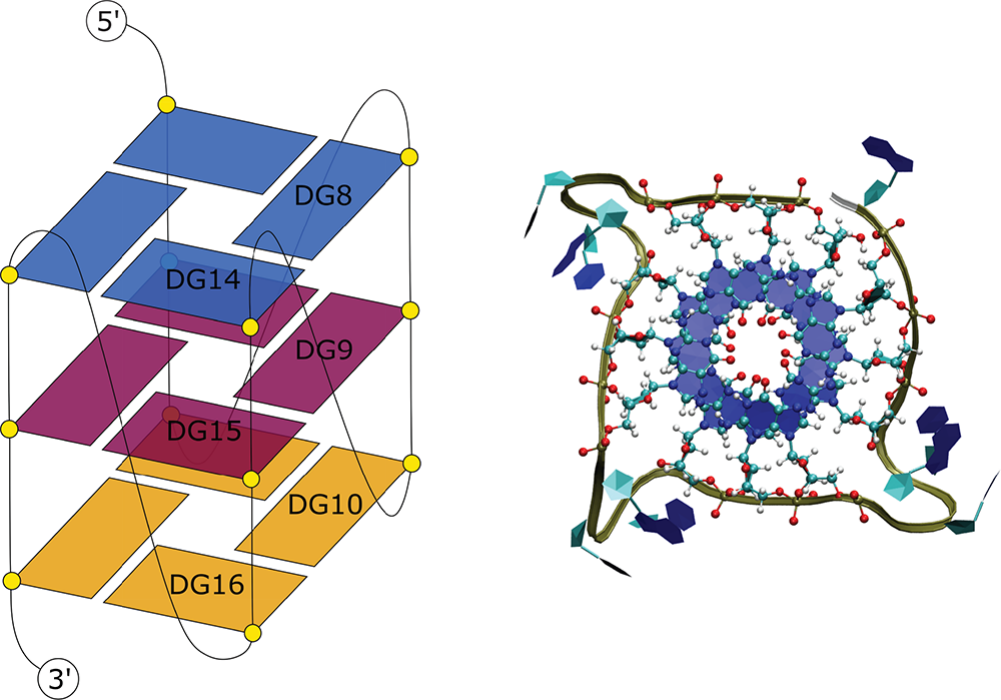
Left: Schematic view of the G-quadruplex scaffold. Right: Top view of the G-quadruplex structure (in polygons and CKP representation).

Finally, due to the supposed role played by guanine as the electron sink in telomeres—DNA tracts rich in guanine—its oxidation potential was calculated in a G-quadruplex 5′-d(A(GGGTTA)_3_GGG)-3′ (GQ). To this end, we calculated the *E*^0^ for a subset of representative guanine bases of GQ applying the PMM–MD procedure. We found a remarkable variation of the *E*^0^ along the sequence: the guanines forming the central plane of the quadruplex show a limited propensity to oxidation (DG9 and DG15 in Table 2). On the contrary, the value of *E*^0^ decreases up to 0.40 V when the guanine belonging to the lower plane of the quadruplex is considered (DG16 in Table 2). Very interestingly, the low values of *E*^0^ observed for guanine located in the external planes strongly suggest that these kinds of constructs can act as electron sinks, thus protecting the cells from oxidants. In fact, such potential G-quadruplex sequences (Figure 3) are often found in 5′-untranslated regions and in the first intron of many genes.^57^

**Table 2:** Calculated Values of the Reduction Potentials of the Guanine Bases in the G-Quadruplex Structure

base	*E*^0^ (V),^a^^,^^b^PMM
DG8	0.58
DG9	0.72
DG10	0.52
DG14	0.54
DG15	0.94
DG16	0.40

a The calculated values are reported against SHE.

b The estimated standard error on the calculated reduction potentials is ±0.04 V.

## Conclusions

We estimated the oxidation potentials of guanine bases on different DNA molecules, single-stranded and double-stranded DNA and G-quadruplex, by means of a theoretical–computational procedure able to include the structural-dynamical effects. Our results, in agreement with experimental data, clearly show that the guanine oxidation potential is influenced by the presence of guanine stretches, which stabilize the oxidized, cationic form of the nucleobase. Such an effect is particularly relevant when the DNA conformation allows a proper interaction between the guanine bases, as occurring at low temperatures in the single-stranded DNA as well as in double-stranded DNA and in G-quadruplex. The stabilizing effect of the presence of consecutive guanines (≈0.10 V for each additional consecutive guanine added to the DNA strand) is quantitatively in line with experimental estimates and, in the case of the G-quadruplex, reaches a remarkably lower value which might explain the cathodic protective effects of this kind of G-rich sequence, particularly present in important gene regulatory regions.

## Supporting Information Available

Details of the calculations, free energy convergence, and analysis of the environment perturbation and DNA charge distribution (PDF)

## References

[ref1] De Bont R.; van Larebeke N. Endogenous DNA damage in humans: A review of quantitative data. Mutagenesis 2004, 19, 169–185. 10.1093/mutage/geh025. 15123782

[ref2] Steel G. G. From targets to genes: a brief history of radiosensitivity. Phys. Med. Biol. 1996, 41, 205–222. 10.1088/0031-9155/41/2/001. 8746105

[ref3] Kawanishi S.; Hiraku Y.; Oikawa S. Mechanism of guanine-specific DNA damage by oxidative stress and its role in carcinogenesis and aging. Mutat. Res. Rev. Mutat. Res. 2001, 488, 65–76. 10.1016/S1383-5742(00)00059-4. 11223405

[ref4] Evans M. D.; Cooke M. S. Factors contributing to the outcome of oxidative damage to nucleic acids. Bioessays 2004, 26, 533–542. 10.1002/bies.20027. 15112233

[ref5] Wohlgamuth C. H.; McWilliams M. A.; Slinker J. D. DNA as a molecular wire: Distance and sequence dependence. Anal. Chem. 2013, 85, 8634–8640. 10.1021/ac401229q. 23964773

[ref6] Berlin Y. A.; Burin A. L.; Ratner M. A. DNA as a molecular wire. Superlattices Microstruct. 2000, 28, 241–252. 10.1006/spmi.2000.0915.

[ref7] Arnold A. R.; Grodick M. A.; Barton J. K. DNA Charge Transport: from Chemical Principles to the Cell. Cell Chem. Biol. 2016, 23, 183–197. 10.1016/j.chembiol.2015.11.010. 26933744 PMC4803044

[ref8] Seidel C. A.; Schulz A.; Sauer M. H. Nucleobase-specific quenching of fluorescent dyes. 1. Nucleobase one-electron redox potentials and their correlation with static and dynamic quenching efficiencies. J. Phys. Chem. 1996, 100, 5541–5553. 10.1021/jp951507c.

[ref9] Steenken S. Purine bases, nucleosides, and nucleotides: Aqueous solution redox chemistry and transformation reactions of their radical cations and e-and OH adducts. Chem. Rev. 1989, 89, 503–520. 10.1021/cr00093a003.

[ref10] Fukuzumi S.; Miyao H.; Ohkubo K.; Suenobu T. Electron-transfer oxidation properties of DNA bases and DNA oligomers. J. Phys. Chem. A 2005, 109, 3285–3294. 10.1021/jp0459763. 16833661

[ref11] D’Abramo M.; Aschi M.; Amadei A. Charge transfer equilibria of aqueous single stranded DNA. Phys. Chem. Chem. Phys. 2009, 11, 10614–10618. 10.1039/b915312h. 20145806

[ref12] D’Abramo M.; Orozco M.; Amadei A. Effects of local electric fields on the redox free energy of single stranded DNA. Chem. Commun. 2011, 47, 2646–2648. 10.1039/C0CC04352D. 21180762

[ref13] Slavicek P.; Winter B.; Faubel M.; Bradforth S. E.; Jungwirth P. Ionization energies of aqueous nucleic acids: Photoelectron spectroscopy of pyrimidine nucleosides and ab initio calculations. J. Am. Chem. Soc. 2009, 131, 6460–6467. 10.1021/ja8091246. 19374336

[ref14] Pluharova E.; Jungwirth P.; Bradforth S. E.; Slavicek P. Ionization of purine tautomers in nucleobases, nucleosides, and nucleotides: from the gas phase to the aqueous environment. J. Phys. Chem. B 2011, 115, 1294–1305. 10.1021/jp110388v. 21247073

[ref15] Paukku Y.; Hill G. Theoretical determination of one-electron redox potentials for DNA bases, base pairs, and stacks. J. Phys. Chem. A 2011, 115, 4804–4810. 10.1021/jp201281t. 21500846

[ref16] Psciuk B. T.; Lord R. L.; Munk B. H.; Schlegel H. B. Theoretical determination of one-electron oxidation potentials for nucleic acid bases. J. Chem. Theory Comput. 2012, 8, 5107–5123. 10.1021/ct300550x. 26593200

[ref17] Lewis K.; Copeland K.; Hill G. One-electron redox properties of DNA nucleobases and common tautomers. Int. J. Quantum Chem. 2014, 114, 1678–1684. 10.1002/qua.24745.

[ref18] Thapa B.; Schlegel H. B. Calculations of p K a’s and redox potentials of nucleobases with explicit waters and polarizable continuum solvation. J. Phys. Chem. A 2015, 119, 5134–5144. 10.1021/jp5088866. 25291241

[ref19] Close D. M.; Wardman P. Calculations of the Energetics of Oxidation of Aqueous Nucleosides and the Effects of Prototropic Equilibria. J. Phys. Chem. A 2016, 120, 4043–4048. 10.1021/acs.jpca.6b02653. 27219530

[ref20] Kumar A.; Adhikary A.; Sevilla M. D.; Close D. M. One-electron oxidation of ds (5′-GGG-3′) and ds (5′-G (8OG) G-3′) and the nature of hole distribution: A density functional theory (DFT) study. Phys. Chem. Chem. Phys. 2020, 22, 5078–5089. 10.1039/C9CP06244K. 32073006 PMC7058519

[ref21] Bravaya K. B.; Epifanovsky E.; Krylov A. I. Four bases score a run: Ab initio calculations quantify a cooperative effect of H-bonding and π-stacking on the ionization energy of adenine in the AATT tetramer. J. Phys. Chem. Lett. 2012, 3, 2726–2732. 10.1021/jz3011139. 26295899

[ref22] Pluharova E.; Slavicek P.; Jungwirth P. Modeling photoionization of aqueous DNA and its components. Acc. Chem. Res. 2015, 48, 1209–1217. 10.1021/ar500366z. 25738773

[ref23] Tóth Z.; Kubečka J.; Muchová E.; Slavíček P. Ionization energies in solution with the QM: QM approach. Phys. Chem. Chem. Phys. 2020, 22, 10550–10560. 10.1039/C9CP06154A. 32010902

[ref24] Miertuš S.; Scrocco E.; Tomasi J. Electrostatic interaction of a solute with a continuum. A direct utilizaion of AB initio molecular potentials for the prevision of solvent effects. Chem. Phys. 1981, 55, 117–129. 10.1016/0301-0104(81)85090-2.

[ref25] Cossi M.; Barone V. Solvent effect on vertical electronic transitions by the polarizable continuum model. J. Chem. Phys. 2000, 112, 2427–2435. 10.1063/1.480808.

[ref26] Marenich A. V.; Cramer C. J.; Truhlar D. G. Universal solvation model based on solute electron density and on a continuum model of the solvent defined by the bulk dielectric constant and atomic surface tensions. J. Phys. Chem. B 2009, 113, 6378–6396. 10.1021/jp810292n. 19366259

[ref27] Close D. M.; Crespo-Hernández C. E.; Gorb L.; Leszczynski J. Influence of microhydration on the ionization energy thresholds of thymine: Comparisons of theoretical calculations with experimental values. J. Phys. Chem. A 2006, 110, 7485–7490. 10.1021/jp061064k. 16759139

[ref28] Close D. M.; Crespo-Hernández C. E.; Gorb L.; Leszczynski J. Theoretical elucidation of conflicting experimental data on vertical ionization potentials of microhydrated thymine. J. Phys. Chem. A 2008, 112, 4405–4409. 10.1021/jp711157b. 18402430

[ref29] Aschi M.; Spezia R.; Di Nola A.; Amadei A. A first-principles method to model perturbed electronic wavefunctions: the effect of an external homogeneous electric field. Chem. Phys. Lett. 2001, 344, 374–380. 10.1016/S0009-2614(01)00638-8.

[ref30] Spezia R.; Aschi M.; Di Nola A.; Amadei A. Extension of the perturbed matrix method: application to a water molecule. Chem. Phys. Lett. 2002, 365, 450–456. 10.1016/S0009-2614(02)01449-5.

[ref31] Zanetti-Polzi L.; Del Galdo S.; Daidone I.; D’Abramo M.; Barone V.; Aschi M.; Amadei A. Extending the perturbed matrix method beyond the dipolar approximation: comparison of different levels of theory. Phys. Chem. Chem. Phys. 2018, 20, 24369–24378. 10.1039/C8CP04190C. 30215645

[ref32] D’Annibale V.; Nardi A. N.; Amadei A.; D’Abramo M. Theoretical characterization of the reduction potentials of nucleic acids in solution. J. Chem. Theory Comput. 2021, 17, 1301–1307. 10.1021/acs.jctc.0c00728. 33621084 PMC8028051

[ref33] Saito I.; Nakamura T.; Nakatani K.; Yoshioka Y.; Yamaguchi K.; Sugiyama H. Mapping of the hot spots for DNA damage by one-electron oxidation: efficacy of GG doublets and GGG triplets as a trap in long-range hole migration. J. Am. Chem. Soc. 1998, 120, 12686–12687. 10.1021/ja981888i.

[ref34] Cauët E. Unique hole-trapping property of the human telomere sequence. J. Biomol. Struct. Dyn. 2011, 29, 557–561. 10.1080/07391102.2011.10507405. 22066540

[ref35] Capobianco A.; Caruso T.; D’Ursi A. M.; Fusco S.; Masi A.; Scrima M.; Chatgilialoglu C.; Peluso A. Delocalized hole domains in guanine-rich DNA oligonucleotides. J. Phys. Chem. B 2015, 119, 5462–5466. 10.1021/acs.jpcb.5b02940. 25839102

[ref36] Conwell E.; Basko D. Hole traps in DNA. J. Am. Chem. Soc. 2001, 123, 11441–11445. 10.1021/ja015947v. 11707121

[ref37] Jin Y.; Ru X.; Su N. Q.; Mei Y.; Beratan D. N.; Zhang P.; Yang W. Revisiting the Hole Size in Double Helical DNA with Localized Orbital Scaling Corrections. J. Phys. Chem. B 2020, 124, 3428–3435. 10.1021/acs.jpcb.0c03112. 32272019 PMC7456463

[ref38] Kurnikov I.; Tong G.; Madrid M.; Beratan D. Hole size and energetics in double helical DNA: competition between quantum delocalization and solvation localization. J. Phys. Chem. A 2002, 106, 7–10. 10.1021/jp0132329.

[ref39] Voityuk A. A. Charge transfer in DNA: Hole charge is confined to a single base pair due to solvation effects. J. Chem. Phys. 2005, 122, 204904 10.1063/1.1924551. 15945774

[ref40] Voityuk A. A. Are radical cation states delocalized over GG and GGG hole traps in DNA?. J. Phys. Chem. B 2005, 109, 10793–10796. 10.1021/jp050985c. 16852312

[ref41] Blancafort L.; Voityuk A. A. CASSCF/CAS-PT2 study of hole transfer in stacked DNA nucleobases. J. Phys. Chem. A 2006, 110, 6426–6432. 10.1021/jp061184s. 16706397

[ref42] Kumar A.; Sevilla M. D. Density functional theory studies of the extent of hole delocalization in one-electron oxidized adenine and guanine base stacks. J. Phys. Chem. B 2011, 115, 4990–5000. 10.1021/jp200537t. 21417208 PMC3084348

[ref43] Adhikary A.; Khanduri D.; Sevilla M. D. Direct observation of the hole protonation state and hole localization site in DNA-oligomers. J. Am. Chem. Soc. 2009, 131, 8614–8619. 10.1021/ja9014869. 19469533 PMC2735011

[ref44] Amadei A.; Daidone I.; Bortolotti C. A. A general statistical mechanical approach for modeling redox thermodynamics: the reaction and reorganization free energies. RSC Adv. 2013, 3, 19657–19665. 10.1039/c3ra42842g.

[ref45] Zanetti-Polzi L.; Bortolotti C. A.; Daidone I.; Aschi M.; Amadei A.; Corni S. A few key residues determine the high redox potential shift in azurin mutants. Org. Biomol. Chem. 2015, 13, 11003–11013. 10.1039/C5OB01819F. 26381463

[ref46] Amadei A.; Daidone I.; Aschi M. A general theoretical model for electron transfer reactions in complex systems. Phys. Chem. Chem. Phys. 2012, 14, 1360–1370. 10.1039/C1CP22309G. 22158942

[ref47] Del Galdo S.; Aschi M.; Amadei A. In silico characterization of bimolecular electron transfer reactions: The ferrocene–ferrocenium reaction as a test case. Int. J. Quantum Chem. 2016, 116, 1723–1730. 10.1002/qua.25212.

[ref48] Frisch M. J.; Trucks G. W.; Schlegel H. B.; Scuseria G. E.; Robb M. A.; Cheeseman J. R.; Scalmani G.; Barone V.; Petersson G. A.; Nakatsuji H.; Gaussian 16, Revision C.01; Gaussian Inc.: Wallingford, CT, 2016.

[ref49] Aidas K.; et al. The Dalton quantum chemistry program system. WIREs Comput. Mol. Sci. 2014, 4, 269–284. 10.1002/wcms.1172. PMC4171759 25309629

[ref50] Berendsen H. J.; Postma J. P.; van Gunsteren W. F.; Hermans J. Interaction models for water in relation to protein hydration. Intermolecular forces 1981, 14, 331–342. 10.1007/978-94-015-7658-1_21.

[ref51] Espinoza E. M.; Clark J. A.; Soliman J.; Derr J. B.; Morales M.; Vullev V. I. Practical Aspects of Cyclic Voltammetry: How to Estimate Reduction Potentials When Irreversibility Prevails. J. Electrochem. Soc. 2019, 166, H3175–H3187. 10.1149/2.0241905jes.

[ref52] Li K.; Yatsunyk L.; Neidle S. Water spines and networks in G-quadruplex structures. Nucleic Acid Res. 2021, 49, 519–528. 10.1093/nar/gkaa1177. 33290519 PMC7797044

[ref53] Bussi G.; Donadio D.; Parrinello M. Canonical sampling through velocity rescaling. J. Chem. Phys. 2007, 126, 014101 10.1063/1.2408420. 17212484

[ref54] Abraham M. J.; Murtola T.; Schulz R.; Páll S.; Smith J. C.; Hess B.; Lindahl E. GROMACS: High performance molecular simulations through multi-level parallelism from laptops to supercomputers. SoftwareX 2015, 1, 19–25. 10.1016/j.softx.2015.06.001.

[ref55] Langley D. R. Molecular dynamic simulations of environment and sequence dependent DNA conformations: the development of the BMS nucleic acid force field and comparison with experimental results. J. Biomol. Struct. Dyn. 1998, 16, 487–509. 10.1080/07391102.1998.10508265. 10052609

[ref56] Isse A. A.; Gennaro A. Absolute potential of the standard hydrogen electrode and the problem of interconversion of potentials in different solvents. J. Phys. Chem. B 2010, 114, 7894–7899. 10.1021/jp100402x. 20496903

[ref57] Fleming A. M.; Burrows C. J. Interplay of Guanine Oxidation and G-Quadruplex Folding in Gene Promoters. J. Am. Chem. Soc. 2020, 142, 1115–1136. 10.1021/jacs.9b11050. 31880930 PMC6988379

