# Supplementary material for: Rationalizing Sequence and Conformational Effects on the Guanine Oxidation in Different DNA Conformations
Source: J Phys Chem B. 2022 Jun 7;126(27):5017–23. doi: 10.1021/acs.jpcb.2c02391 (PMC9289878; doi:10.1021/acs.jpcb.2c02391)
Supplement: Supplementary file 1 — jp2c02391_si_001.pdf [file jp2c02391_si_001.pdf]

Supplementary Information: Rationalizing Sequence and Conformational effects on the guanine oxidation in different DNA conformations

Alessandro Nicola Nardi, Alessio Olivieri, and Marco D'Abramo*

Department of Chemistry, Sapienza University of Rome, Rome, Italy

E-mail: marco.dabramo@uniroma1.it

Directions of perturbing electric field in ss-HG1 and ds-HG1 systems

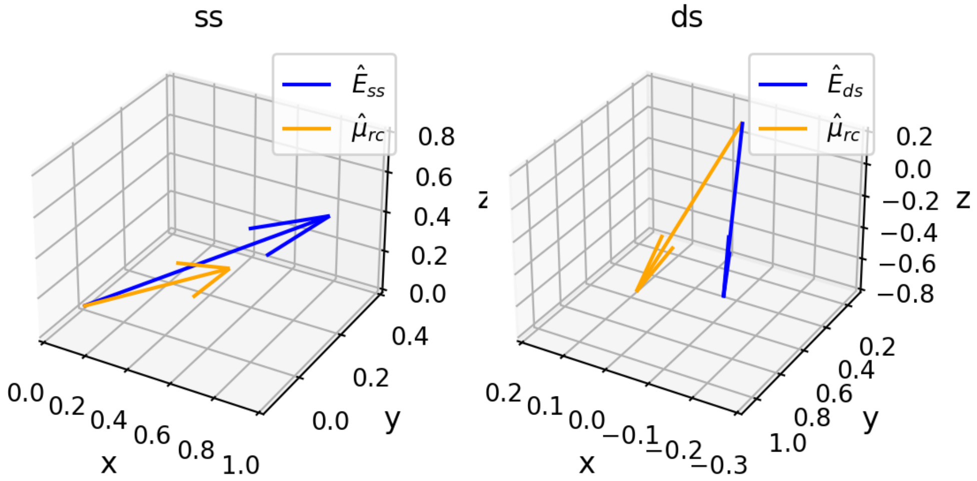

Figure S1: Direction of the dipole moment of the ionized guanine ($\hat{\mu}_{rc}$) in the ss-HG1 and ds-HG1 substrates (left and right panel, respectively), in yellow. Mean directions of the electric field generated by the ss-HG1 and ds-HG1 ($\hat{E}_{ss}$ left panel; $\hat{E}_{ds}$ right panel) structures on the center of mass of the ionized guanine, in blue. The reference systems of the MD simulations were used and the versors were translated to the origin.

Geometry and spin density distribution of the ionized guanine–guanine dimer, $[GG]^{\cdot+}$

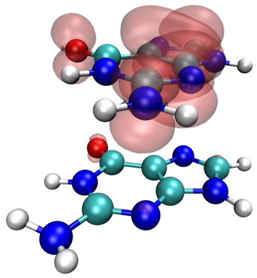

Figure S2: Ionized guanine–guanine dimer and its calculated spin density distribution (CAM-B3LYP).

Charge distributions of $[GG]^{\cdot+}$ dimer in gas phase and in water (PCM)

Table S1: Charge distribution of the ideal stacked cationic guanine–guanine dimer in gas phase and in water (as modelled by PCM).

Base	B3LYP	CAM-B3LYP	M062X	MP2
gas phase				
5' G	0.73	0.85	0.84	0.90
3' G	0.27	0.15	0.16	0.10
water (PCM)				
5' G	0.87	0.96	0.95	0.97
3' G	0.13	0.04	0.05	0.03

Free energy convergence

Reduction free energy (ΔA) convergence was checked, in both neutral and oxidized ensemble, calculating ΔA as a function of the number of MD trajectory frames used:

$$\Delta A(n) \simeq \frac{k_B T}{2} \ln \frac{\langle e^{-\beta \Delta \mathcal{U}_e} \rangle_{red}}{\langle e^{\beta \Delta \mathcal{U}_e} \rangle_{ox}} = \frac{k_B T}{2} \ln \frac{\sum_{i=1}^{n} e^{-\beta \Delta \mathcal{U}_{e,red}(n)}}{\sum_{i=1}^{n} e^{\beta \Delta \mathcal{U}_{e,ox}(n)}}$$
$$= \frac{1}{2} \left[k_B T \ln \sum_{i=1}^{n} e^{-\beta \Delta \mathcal{U}_{e,red}(n)} + k_B T \ln \sum_{i=1}^{n} e^{\beta \Delta \mathcal{U}_{e,ox}(n)} \right] \tag{S1}$$

In Eq. S1, $\Delta \mathcal{U}_{e,red} = \Delta \mathcal{U}_{e,red}(n)$ and $\Delta \mathcal{U}_{e,ox} = \Delta \mathcal{U}_{e,ox}(n)$ are the electronic energy differences between the oxidized and neutral guanine base in the reduced and oxidized ensemble at the n-th MD frame, respectively.

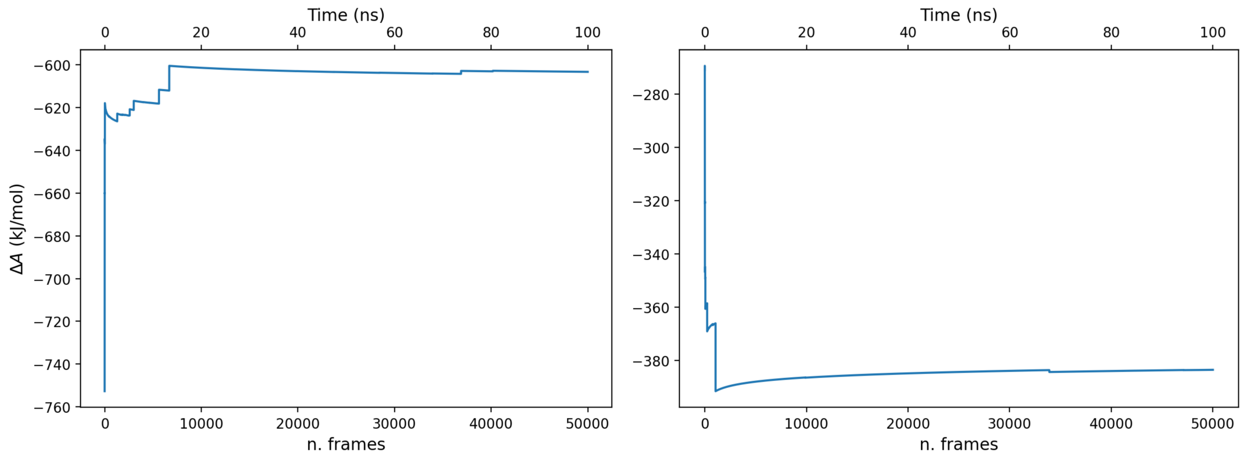

Figure S3: Reduction free energy of ionized guanine in ss-HG1 as a function of the number of frames (bottom axis) and time (upper axis). Right panel: neutral ensemble (first term in square brackets in Eq. S1). Left panel: oxidized ensemble (second term in square brackets in Eq. S1).

Similar trends were observed for the other systems, suggesting that 100 ns were sufficient to achieve the convergence of ΔA.

Helical parameters of ss-HG2 at different temperatures

Table S2: Standard deviations of the typical DNA parameters as provided by MD simulations of the ss-HG2 at different temperatures.

Parameter		σ	σ
		$278K$	$300K$
Shift	Å	1.49	2.32
Slide	Å	0.78	1.09
Rise	Å	0.26	0.49
Tilt	deg.	4.36	8.46
Roll	deg.	9.02	16.07
Twist	deg.	12.75	23.20

Reduction potential of $G^{\cdot+}$ without the perturbing contribution of the neighbour nucleobase

Table S3: Calculated values of the cationic guanine ($G^{\cdot+}$) reduction potentials in the ds-HG1 and ds-HG2 DNA without the perturbing contribution of the neighbour nucleobase in 5' direction.

System	$T(K)$	$E^0(V)^{a,b}$
		PMM
ds-HG1	278	1.10
ds-HG2	278	1.40

[a]Values are reported against SHE. [b]The estimated standard error on the calculated reduction potentials is ± 0.04 V.
